# The association between patients' illness perceptions and longitudinal clinical outcome in patients with low back pain

**DOI:** 10.1097/PR9.0000000000001004

**Published:** 2022-04-27

**Authors:** Maria Fors, Birgitta Öberg, Paul Enthoven, Karin Schröder, Allan Abbott

**Affiliations:** a Unit of Physiotherapy, Department of Health, Medicine and Caring Sciences, Linköping University, Linköping, Sweden; b Department of Activity and Health in Linköping, and Department of Health, Medicine and Caring Sciences, Linköping University, Linköping, Sweden

**Keywords:** Low back pain, Illness perceptions, Prospective cohort, Common-Sense Model of Self-Regulation, Patient-reported outcome, Clinical outcomes

## Abstract

**Introduction:**

Illness perception is suggested to influence outcome in patients with low back pain (LBP). It is unknown if specific illness perceptions are of more importance for longitudinal outcomes, including development of self-management strategies.

**Objectives:**

This study explores whether patients' initial illness perceptions were associated with disability, pain, health-related quality of life, and self-care enablement outcomes in patients with LBP after 3 and 12 months.

**Methods:**

Four hundred sixty-seven consecutive patients seeking physiotherapeutic primary care for LBP were eligible to participate in this prospective cohort study, providing data at baseline and after 3 and 12 months (mean age 45 years, 56% women). Multiple linear regression analysis was used to explore whether patients' illness perceptions at baseline were associated with outcome in the Oswestry Disability Index (ODI), Numeric Rating Scale–LBP (NRS-LBP), EuroQol Five Dimensions, and Patient Enablement Instrument (PEI).

**Results:**

Stronger beliefs that the back problem will last a long time at baseline were associated with worse outcome in ODI, NRS-LBP, and PEI at 3 and 12 months and in EuroQol Five Dimensions at 12 months. Negative beliefs regarding treatment's ability to improve LBP were associated with worse outcome in NRS-LBP and PEI at 3 and 12 months and in ODI at 12 months.

**Conclusions:**

Illness perceptions regarding prognosis and treatment's ability to improve symptoms were the most prominent perceptions explaining several longitudinal clinical outcomes. These expectations should be addressed in an early stage in the delivery of interventions for LBP. These expectations were also important for patients' development of coping and self-management strategies.

## Abstract 2

Initial perceptions regarding prognosis and treatment effects were prominent perceptions explaining longitudinal outcomes in patients with low back pain, including explaining patients' development of self-management strategies.

## 1. Introduction

The identification of key prognostic factors is important to identify patients with low back pain (LBP) at risk of poor outcomes.^23,40^ Treatment guidelines put emphasis on identifying psychological obstacles for recovery and recommend their assessment when treating patients with LBP.^10,21,43^ Illness perceptions, which are individuals' beliefs about their condition, have received increasing attention because of their suggested influence on outcome in patients with LBP.^23^ Furthermore, illness perceptions are purported to influence illness outcome within the Common-Sense Model of Self-Regulation (CSM).^35^ The model suggests that people develop their own set of beliefs and ideas about their illness (illness representations). These illness representations are divided into emotional and cognitive representations, where the cognitive representations are categorized into several dimensions. The model suggests that emotional and cognitive illness representations have an impact on emotional responses and behavior influencing coping strategies and action plans, for example, creating and implementing self-management strategies. This may in turn affect illness outcomes and emotional well-being.^35^

Development of tools designed to inform decisions about appropriate care require an initial understanding of what factors are associated with longitudinal outcome and are most likely to be modified by interventions.^23,40^ Illness perceptions have in comparison with other psychological constructs been shown to have stronger associations with clinical outcomes at both short- and long-term follow-up in patients with LBP.^7,23,38^ Illness perceptions have been shown to change over time for those patients with LBP who recover.^22^ Furthermore, study results also indicate that LBP-related illness perceptions can be modified by interventions targeting specific illness perceptions.^39,47^ However, only few studies have investigated if there are specific baseline illness perceptions of more importance for longitudinal outcomes in patients with LBP.^7,22–24^ These studies have reported some similar LBP illness perceptions having associations with pain and disability, such as perceptions relating to prognosis and controllability over the back problem.^7,22–24^

Health-related quality of life (HRQoL), together with pain- and disability-related outcomes, is in line with core outcome domains recommended to use when evaluating interventions for patients with LBP.^9^ There is a paucity of research investigating if LBP illness perceptions are associated with other longitudinal outcomes apart from pain- and disability-related outcomes. For example, only few studies have included HRQoL-related longitudinal outcomes and they found an association with baseline illness perceptions in patients with LBP.^24,38^ Further, LBP treatment guidelines recommend interventions aiming to empower patients to self-manage their back pain. The CSM suggests that illness perceptions may influence individuals' self-management of ongoing and future illness, for example, through the choice of coping strategies.^35^ There are no studies investigating the relationship between illness perceptions and patient enablement, which represent patients' understanding of and coping with illness.^30,31^ The aim of this study was to explore whether patients' initial illness perceptions are associated with disability, pain, HRQoL, and self-care enablement outcomes in patients with LBP at 3 and 12 months after seeking physiotherapeutic primary care. No hypothesis was predefined.

## 2. Methods

### 2.1. Design and settings

The current study applied a prospective cohort design in an exploratory analysis of data from a parent study with an experimental design in the form of a cluster randomized controlled trial. The parent study has a published a priori protocol^1^ and is also registered on clinicaltrials.gov (NCT03147300). The cohort consisted of patients with LBP seeking care at 15 public financed primary care physiotherapy rehabilitation clinics in South-East Sweden between April 2017 and March 2018. Patients were consecutively recruited by physiotherapists working at the rehabilitation clinics. All patients received physiotherapy care. Ethical approval for the study was obtained from the Regional Ethics Committee in Linköping (Approval number: Dnr 2017-35/31). Informed consent was obtained from all patients involved in the study.

### 2.2. Participants

The study included 467 patients in the age of 18 to 65 years, fluent in Swedish, and accessed public primary care because of a first-time or recurrent episode of acute-, subacute-, or chronic-phase benign LBP with or without radiculopathy. Exclusion criteria were current diagnosis of malignancy, spinal fracture, infection, cauda equina syndrome, ankylosing spondylitis or systemic rheumatic disease, and previous malignancy during the past 5 years; spinal surgery during the last 2 years; current pregnancy or previous pregnancy up to 3 months before consideration of inclusion; patients who fulfilled the criteria for multimodal/multiprofessional rehabilitation for complex long-standing pain; and severe psychiatric diagnosis. The sample size was based on the parent study that was powered to detect a between-group change with an effect size of *d* = 0.35 at 80% power and a 1-tailed *P* = 0.05 for an a priori hypothesized superiority of treatment according to the BetterBack Model of Care compared with routine care. The results, however, rejected the a priori hypothesis based on no statistically significant difference in patient-reported outcomes.^46^

### 2.3. Outcomes

Patient-reported outcome measures (PROMs) and demographic information were obtained during the first visit to the physiotherapist. Postal questionnaires were sent to the patients 3 and 12 months after the first visit. The PROMs included are described below.

The Oswestry Disability Index (ODI) was used to assess disability. Oswestry Disability Index is a valid LBP-specific measure of pain-related function and activity limitations presented as a score ranging between 0% and 100% disability.^18,19^ The ODI has been found to be valid and reliable for use in patients with LBP in Scandinavian countries.^26,34^

The Numeric Rating Scale for lower back–related pain intensity (NRS-LBP) was used to rate pain intensity on a numerical scale ranging from 0 (no pain) to 10 (worst pain imaginable).^12,32^

The EuroQol Five Dimensions (EQ-5D) was used to assess HRQoL. The EQ-5D covers 5 dimensions of health: self-care, pain or discomfort, anxiety or depression, mobility, and usual activities. The scores create an index ranging from −0.594 to 1, where 1 indicates optimal health.^17^ The ODI, NRS, and EQ-5D cover core outcome domains recommended for the evaluation of clinical trials on LBP.^9^

The Patient Enablement Instrument (PEI) was used to assess patients' self-perceived ability to understand and cope with illness^45^ and can be considered a proxy for self-care enablement.^30,31^ The total score ranges from 0 to 12, where higher scores indicate better/more enablement.^45^ The Swedish version of PEI has shown to be a valid and reliable instrument for use in patients with musculoskeletal pain^15^ and in primary care setting^45^ in Sweden.

Patients' illness perceptions were assessed with the Brief Illness Perception Questionnaire (BIPQ), which has been developed based on the CSM.^35^ The questionnaire includes 9 items comprising cognitive and emotional illness representations specified in the CSM. Eight items are assessed on a scale from 0 to 10 with endpoint descriptors. A higher score reflects a more threatening view of the illness. The overall total score of all items or subscore for each item can be used. The ninth item, used for categorical analysis, was not collected. Five items in the questionnaire assess cognitive illness representations: *consequences* (“How much does you LBP affect your life? Not affect at all–Severely affects my life”), *timeline* (“How long do you think your LBP will continue? A very short time–Forever”), *personal control* (“How much control do you feel you have over your LBP? Absolutely no control–Extreme amount of control”), *treatment control* (“How much do you think your treatment can help your LBP? Not at all–Extremely helpful”), and *identity* (“How much do you experience symptoms from your LBP? No symptoms at all–Many severe symptoms”). One item assesses *coherence* (“How well do you feel you understand your LBP? Don't understand at all–Understand very clearly”). Two items assess emotional representation: *concern* (“How concerned are you about your LBP? Not at all concerned–Extremely concerned”) and *emotional representation* (“How much does your LBP affect you emotionally? eg, Does it make you angry, scared, upset or depressed? Not at all affected emotionally–Extremely affected emotionally”).^5^ The BIPQ has shown to be a valid and reliable instrument for use in several illness groups.^5,6^ The Norwegian version of the BIPQ has been tested in a sample of patients with LBP and showed good validity and reliability.^37^ The linguistic and cultural similarities between the Scandinavian countries support the use of a Swedish version, suggesting no need for further validation.

### 2.4. Statistical analysis

Descriptive statistics summarizing baseline characteristics and outcome measures were presented in frequency/proportion for categorical variables and mean ± SD for continuous variables. Multiple linear regression analyses were used to explore whether patients' illness perceptions (independent variables) are associated with longitudinal outcome in disability, back pain intensity, HRQoL, and self-care enablement at 3 and 12 months of follow-up (dependent variables). The regression models were adjusted for demographic variables (age and sex) and clinical variables (duration of current episode and baseline score on the dependent variable). The demographic and clinical variables were selected based on their known association with longitudinal outcome in patients with LBP.^8,16,41,49^ The independent variables were entered block wise. The 8 illness perception dimensions from the BIPQ were entered in the first block. Demographic variables and clinical variables were entered in the second block. The data assumptions required for linear regression modelling were tested and confirmed.

Missing data in the PROMs at follow-ups were handled through multiple imputation, based on group data from baseline as well as 3, 6, and 12 months. Pooled data of 100 imputation sets were used for each of the PROMs.^3^ The analyses on the PEI were conducted on Per-Protocol data because the PEI is a transition rating scale only assessed at longitudinal time points and not at baseline and therefore the multiple imputation method could not be used. The sample size of 467 patients was sufficient to detect small effect sizes (*f*^2^ = 0.02) for the associations between the 12 independent variables (13 *df*) and the dependent variables in the multiple linear regression analyses with a 2-tailed significance level set to 0.05 and post-hoc power of 86%.^20,25^ The number of subjects per variable in the current study was 36, which minimized the relative bias in the estimated *R*^2^ and provides accurate estimation of regression coefficients.^2^ Statistical analysis was performed using IBM SPSS statistics version 27.

## 3. Results

Of the 1034 potentially eligible patients seeking physiotherapy care for LBP, 500 fulfilled the inclusion criteria and accepted participation in the study. Baseline PROMs were attained for 467 patients. The 467 patients had a mean (SD) age of 45.2 (12.2) years and 56% were women. A summary of baseline and prospective data is presented in Table 1. Response to questionnaires after 3 months was 73% (n = 342) and after 12 months was 60% (n = 279) of the included patients.

**Table 1:** Summary of patients' characteristics and self-reported outcomes at baseline and follow-ups (n = 467).

Variables	Baseline	3-month follow-up	12-month follow-up
Age, mean (SD), y	45.2 (12.2)		
Sex, women, n (%)	261 (56)		
Back pain (NRS-LBP), mean (SD)	6.28 (2.22)	3.65 (2.39)	3.47 (2.39)
Leg pain (NRS), mean (SD)	3.67 (3.27)		
Duration of current episode, n (%)			
<12 wk	269 (58)		
>12 wk	198 (42)		
ODI % disability, mean (SD)	31.0 (15.8)	22.0 (15.2)	19.0 (14.2)
EQ-5D, mean (SD)	0.53 (0.3)	0.66 (0.3)	0.67 (0.3)
PEI, mean (SD)		4.42 (4.0) n = 335	4.97 (4.3) n = 262
BIPQ,* mean (SD)			
Consequences	6.68 (2.2)		
Timeline	5.91 (2.4)		
Personal control	4.17 (2.3)		
Treatment control	7.42 (2.0)		
Identity	6.73 (1.7)		
Concern	6.94 (2.4)		
Coherence	5.47 (2.5)		
Emotional representation	5.72 (2.8)		

* Dimensions are scored on a 0- to 10-point scale, where higher score represents worse LBP perception. Dimensions 3, 4, and 7 are reversed.

BIPQ, Brief Illness Perception Questionnaire; EQ-5D, EuroQol Five Dimensions (−0.594 to 1; higher score represents better health status); ODI, Oswestry Disability Index (0–100; higher score indicates greater disability); NRS-LBP, Numeric Rating Scale—Low Back Pain (0–10; higher score indicates higher pain intensity); PEI, Patient Enablement Instrument (0–12; higher score indicates greater ability to understand and cope with illness).

The results of the multiple linear regression analyses with ODI, NRS-LBP, EQ-5D, and PEI as dependent variables are presented in Table 2. Baseline illness perception dimensions together with demographic and clinical variables explained between 12% (*P* < 0.001) and 34% (*P* < 0.001) of the variance in ODI, NRS-LBP, EQ-5D, and PEI at 3 and 12 months. Baseline illness perception dimensions alone explained between 12% (*P* < 0.001) and 22% (*P* < 0.001) of the variance in ODI, NRS-LBP, EQ-5D, and PEI at 3 and 12 months.

**Table 2:** Associations between illness perceptions and short- and long-term outcome in disability, back pain intensity, health-related quality of life, and self-care enablement using multiple linear regression analyses (n = 467).

	ODI, 3 mo	ODI, 12 mo	NRS-LBP, 3 mo	NRS-LBP, 12 mo	EQ-5D, 3 mo	EQ-5D, 12 mo	PEI, 3 mo, n = 335	PEI, 12 mo, n = 262
Model 1: BIPQ 8 items Adjusted *R*^2^	0.209*	0.220*	0.165*	0.155*	0.132*	0.190*	0.117*	0.215*
Model 2: BIPQ 8 items,demographic† and clinical variables‡ Adjusted *R*^2^	0.337*	0.337*	0.203*	0.208*	0.177*	0.222*	0.119*	0.223*

Independent variables	β (*P*)	β (*P*)	β (*P*)	β (*P*)	β (*P*)	β (*P*)	β (*P*)	β (*P*)
BIPQ								
Consequences								
Timeline	1.134 (0.001)	1.349 (<0.001)	0.151 (0.010)	0.202 (0.001)		−0.021 (0.008)	−0.295 (0.007)	−0.297 (0.013)
Personal control								
Treatment control		0.857 (0.015)	0.141 (0.026)	0.174 (0.009)			−0.312 (0.006)	−0.563 (<0.001)
Identity			0.217 (0.019)				−0.520 (0.001)	
Concern			−0.124 (0.049)					
Coherence								
Emotional representation			0.165 (0.002)		−0.020 (0.002)	−0.016 (0.015)		
Age			−0.022 (0.029)					
Sex: female								
Duration of current episode	3.208 (0.028)	3.742 (0.008)	0.522 (0.039)		−0.063 (0.029)			
Baseline dependent variable	0.455 (<0.001)	0.394 (<0.001)	0.173 (0.004)	0.238 (<0.001)	0.231 (<0.001)	0.149 (0.026)	—	—

Dimensions 3,4 and 7 in the BIPQ have been reversed before analysis. β = unstandardized beta-coefficients.

* *P* < 0.001. Significant associations are presented in the table.

† Sex and age.

‡ Baseline score on the dependent variable, duration of current episode.

BIPQ, Brief Illness Perception Questionnaire Dimensions are scored on a 0- to 10-point scale, where higher score represents worse LBP perception; EQ-5D, EuroQol Five Dimensions (−0.594 to 1; higher score represents better health status); ODI, Oswestry Disability Index (0–100; higher score indicates greater disability); NRS-LBP, Numeric Rating Scale—Low Back Pain (0–10; higher score indicates higher pain intensity); PEI, Patient Enablement Instrument (0–12; higher score indicates greater ability to understand and cope with illness).

Patients' belief that the LBP symptoms will last a long time (*timeline*) at baseline was statistically significantly associated with higher score on the ODI at 3 and 12 months (β = 1.134, *P* = 0.001 and β = 1.349, *P* < 0.001, respectively). Also, more negative beliefs regarding treatment's ability to improve symptoms (*treatment control*) at baseline was statistically significantly associated with higher score on the ODI at 12 months (β = 0.857, *P* = 0.015). Belief that the LBP symptoms will last a long time (*timeline*) and more negative beliefs regarding treatment's ability to improve symptoms (*treatment control*) at baseline were statistically significantly associated with higher score on the NRS-LBP at both 3 months (β = 0.151, *P* = 0.010 and β = 0.141, *P* = 0.026, respectively) and at 12 months (β = 0.202, *P* = 0.001 and β = 0.174, *P* = 0.009, respectively).

Experiencing more and worse symptoms from the lower back (*identity*) and having higher negative emotional response (*emotional representations*) at baseline were statistically significantly associated with higher score on the NRS-LBP at 3 months (β = 0.217, *P* = 0.019 and β = 0.165, *P* = 0.002, respectively). Feeling more concerned regarding the back problem (*concern*) at baseline was significantly associated with lower score on the NRS-LBP at 3 months (β = −0.124, *P* = 0.049). A higher negative emotional response (*emotional representations*) at baseline was statistically significantly associated with lower score on the EQ-5D at both 3 and 12 months (β = −0.020, *P* = 0.002 and β = −0.016, *P* = 0.015, respectively). Further, belief that the LBP symptoms will last a long time (*timeline*) at baseline was statistically significantly associated with lower score on the EQ-5D at 12 months (β = −0.021, *P* = 0.008). Patients' belief that the LBP symptoms will last a long time (*timeline*) and more negative beliefs regarding treatment's ability to improve symptoms (*treatment control*) at baseline were statistically significantly associated with lower score on the PEI at 3 and 12 months (timeline: β = −0.295, *P* = 0.007 and β = −0.297, *P* = 0.013, respectively; treatment control: β = −0.312, *P* = 0.006 and β = −0.563, *P* < 0.001, respectively). Further, experiencing more and worse symptoms from the lower back (*identity*) at baseline was significantly associated with lower score on the PEI at 3 months (β = −0.520, *P* = 0.001).

Among the clinical variables, a worse baseline score on the dependent variable was statistically significantly associated with worse score in all outcome measures at 3 and 12 months. Longer duration of the current LBP episode was statistically significantly associated with worse score on the ODI, NRS-LBP, and EQ-5D at 3 months and worse score on the ODI at 12 months. The demographic variables were not associated with longitudinal outcomes, except that a higher age was significantly associated with lower score on the NRS-LBP at 3 months.

## 4. Discussion

The study results showed, in line with the CSM, that baseline illness perceptions significantly explained variation in both short- and long-term clinical outcomes while controlling for demographic and clinical variables. Among baseline illness perception dimensions, patients' belief that the LBP symptoms will last a long time (*timeline*) was consistently associated with worse outcome in all PROMs, except for the short-term outcome in EQ-5D. Among previous studies investigating different illness perceptions association with longitudinal outcomes in patients with LBP,^7,22–24^ Foster et al.^22^ found that patients' belief that symptoms will last a long time (*timeline*) was associated with poor outcome in pain and disability 6 months after seeking care. The *timeline* illness perception dimension also had the strongest association with longitudinal outcome when compared with 20 psychological constructs in the same study cohort.^23^ In contrast to the current study result, patients who held weak beliefs about controllability of their back problem (*personal control*) and patients who perceived severe consequences of their back problem (*consequences*) at baseline were also seen to have higher risk of poor outcome in pain and disability.^22^ Although, beliefs regarding how long symptoms will last (*timeline*), together with baseline pain intensity, were the only predicting factors of outcome among other illness perception dimensions and other psychological factors at 5-year follow-up in the same cohort.^7^ This supports the current study results that beliefs regarding prognosis (*timeline*) is the prominent LBP illness perception dimension associated with patient-rated longitudinal outcomes.

Our study result showed that various baseline illness perceptions were associated with different clinical outcomes at prospective follow-ups. Adding to previous studies on illness perceptions in the research field of LBP, the current study investigated initial illness perceptions' associations with other longitudinal outcomes besides those related to pain and disability. To our knowledge, only 1 study has compared different illness perceptions in relation with other outcomes than pain and disability in patients with LBP, although the authors did not draw any conclusion about specific illness perceptions.^24^ In line with the current study, the baseline score on the illness perception dimension *emotional representation* (negative emotional reactions to illness) has to a larger extent been associated with longitudinal outcome in HRQoL, whereas *treatment control* (beliefs regarding treatment's ability to improve symptoms) has to a larger extent been associated with longitudinal outcome in disability in other patient groups.^6^ Several baseline illness perception dimensions were associated with back pain intensity at 3 months. More concern regarding the back problem (*concern*) at baseline was associated with lower pain intensity at 3 months, whereas higher negative emotional response (*emotional representation*) at baseline was associated with higher pain intensity at 3 months. These results suggest that patients with a higher degree of concern as an emotional response to the back problem may take more active coping strategies to reduce their back problems, which is supported in previous literature.^48,50^ Such active strategies may be information seeking about their problem or using an expected effective strategy like physical activity. In contrast, a higher degree of depression and anger as an emotional response to the back problem may lead to maladaptive strategies.

Patients with LBP are a heterogenous patient group in several aspects. Multiple factors contribute to the pain and disability in LBP.^28^ This study's population is also heterogenous regarding symptom duration. It is likely that illness perceptions may differ between patients with different symptom duration. Some illness perception dimensions' regression coefficients were stronger or had equally strong multivariate association as those of the baseline score on the dependent variable, but most of the illness perception dimensions had low regression coefficients overall. However, baseline illness perception dimensions *timeline* and *treatment control* had multivariate association with several of the clinical outcomes at prospective follow-ups. This suggests that there may be mutual illness perceptions of importance for clinical outcomes. The illness perception dimensions in the CSM are not independent, yet they will be related in different ways depending on the character of the referred illness.^6,35^ This supports the suggestion that some illness perceptions may have greater influence on outcome. Further, illness perception dimensions *timeline* and *treatment control* are both dimensions in the CSM that have an expectation focus.^35^ The questions measuring these illness perception dimensions in BIPQ clearly capture expectations regarding prognosis and treatment effect: “How long do you think your illness will continue?” and “How much do you think your treatment can help your Illness?” This illustrates the importance of expectations in LBP recovery.

Our study result indicates patients' expectations regarding prognosis and treatment effect to be of importance for longitudinal clinical outcomes in patients with LBP. This is supported by previous literature.^27,29,42^ In qualitative studies, it is found that people with LBP mainly request information on prognosis, treatment options, and self-management strategies besides information on the diagnosis.^36^ Advice and other interventions may not make sense for patients with unhelpful perceptions regarding the prognosis and how suggested treatment will improve their symptoms. This suggests having a dialog with the patient about prognosis and treatment expectations to affect potentially unhelpful perceptions that the patient may hold influencing treatment outcome and to help develop a shared understanding between the clinician and the patient about the back problem.

In the LBP research field, patients' illness perceptions, in general, have been studied in relation to clinical outcomes. Adding to existing literature, the current study investigated illness perceptions potential influence on patients' self-care enablement in developing strategies to understand and cope with their LBP. Treatment guidelines for LBP emphasize enhancing patients' ability to self-manage throughout the entire care process and at all stages in the course of the disease considering the fluctuating character of the condition.^10^ The current study results showed illness perceptions significantly explained variation in both short- and long-term outcome in patients' self-care enablement, although explaining variation in the long-term outcome to a higher extent. Evaluating patients' self-care enablement would reflect “coping appraisal” in the CSM, which is patients' ongoing evaluation of their self-management strategies in the self-regulation process.^35^ Positive beliefs regarding prognosis (*timeline*) and treatment's ability to improve symptoms (*treatment control*) at baseline were significantly associated with better patient self-care enablement at short- and long-term follow-up. According to the CSM, expecting short symptom duration and having high treatment expectations could be interpreted as facilitators for patients developing adequate self-management strategies. To facilitate patients' self-management, patients may need help to make sense of their symptoms, such as their expected prognosis, as well as clarification on how treatment and advised management strategies may lead to improvement in their back problems.

There are strengths and limitations to this study. The study participants had similar characteristics compared with those in other studies in the context of primary care.^4,16,23^ Those patients who did not participate in the study, for example, those who seek other care givers, may hold different perceptions about their back problem. The regression models were adjusted for patients' baseline score in the dependent variable, which have been seen to be associated with longitudinal outcome in patients with LBP.^16^ The regression analyses were also adjusted for duration of the current LBP episode to recognize that patients with different symptom durations at first consultation with the physiotherapist may hold different illness perceptions^23^ and may have different clinical courses.^8^ The 3 and 12 months of follow-up period were set to evaluate short- and long-term clinical outcome. In an average course of LBP, improvement occurs within 6 to 12 weeks, followed by much smaller changes after this period.^11,13,44^ The course of LBP may be fluctuating and the prognosis differ among patients.^14,33^ A longer follow-up period may capture more of the LBP course, although individual fluctuation in the clinical course would still not be captured. All participating patients received the current routine physiotherapy care for LBP for the timepoint when they sought care. The main results from the cluster randomized controlled trial investigating implementation of a physiotherapy model of care (BetterBack) for patients with LBP showed no statistically significant difference in ODI, NRS-LBP, EQ-5D, PEI, and the total score for the BIPQ between patients receiving care before and after the implementation of the model of care.^46^ Thus, no adjustments for treatment according to group allocation were made.

Illness perceptions regarding LBP prognosis (*timeline*) and treatment's ability to improve symptoms (*treatment control*) were shown to be the most prominent perceptions explaining several longitudinal clinical outcomes in patients with LBP. These expectations should be addressed at an early stage in the delivery of interventions for LBP. Even if these 2 illness perception dimensions proved to be important, there is still a significant individual variation of interest in relation to different clinical outcomes. Apart from the influence on clinical outcomes, *timeline* and *treatment control* are also important for patients' development of coping and self-management strategies. To facilitate patients' self-management, patients may need help to understand their expected prognosis and also clarification about how treatment may lead to improvement. Further research should investigate if targeting patients' illness perceptions may generate improved clinical outcomes and interact with self-management development for patients with LBP.

## Disclosures

The authors have no conflicts of interest to declare.This research was funded by Swedish Research Council grant number 2017*01444, Research Council in Southeast Sweden grant number FORSS*660371, FORSS*757721 and grant number FORSS*931966, Region of Östergötland grant number RO-938179 and RO-921021.

The dataset used or analyzed during the current study are available from corresponding author on reasonable request.

Study registry: The cluster randomized controlled trial is registered on clinicaltrials.gov (NCT03147300). The exploratory analyses reported in the present study are not preregistered.
